# The Impact of Yangtze River Discharge, Ocean Currents and Historical Events on the Biogeographic Pattern of *Cellana toreuma* along the China Coast

**DOI:** 10.1371/journal.pone.0036178

**Published:** 2012-04-26

**Authors:** Yun-wei Dong, Hai-shan Wang, Guo-Dong Han, Cai-huan Ke, Xin Zhan, Tomoyuki Nakano, Gray A. Williams

**Affiliations:** 1 State Key Laboratory of Marine Environmental Science, College of Oceanography and Earth Science, Xiamen University, Xiamen, China; 2 Marine Biodiversity and Global Change Laboratory, Xiamen University, Xiamen, China; 3 Seto Marine Biological Laboratory, Field Science Education and Research Centre, Kyoto University, Nishimuro, Wakayama, Japan; 4 The Swire Institute of Marine Science and School of Biological Sciences, The University of Hong Kong, Hong Kong SAR, China; University of Canterbury, New Zealand

## Abstract

**Aim:**

Genetic data were used to measure the phylogeographic distribution of the limpet, *Cellana toreuma* along the China coast in order to acsertain impacts of historic events, ocean currents and especially freshwater discharge from the Yangtze River on the connectivity of intertidal species with limited larval dispersal capability.

**Methodology/Principal Findings:**

Genetic variation in 15 populations of *C. toreuma* (n = 418), ranging from the Yellow Sea (YS), East China Sea (ECS) and South China Sea (SCS), were determined from partial mitochondrial cytochrome c oxidase subunit I gene. Genetic diversity and divergence based on haplotype frequencies were analyzed using CONTRIB, and AMOVA was used to examine genetic population structure. Historic demographic expansions were evaluated from both neutrality tests and mismatch distribution tests. Among the 30 haplotypes identified, a dominant haplotype No. 1 (H1) existed in all the populations, and a relatively abundant private haplotype (H2) in YS. Pairwise F_ST_ values between YS and the other two groups were relatively high and the percentage of variation among groups was 10.9%.

**Conclusions:**

The high nucleotide and gene diversity in the YS, with large pairwise genetic distances and relatively high percentages of variation among groups, suggests that this group was relatively isolated from ECS and SCS. This is likely driven by historic events, ocean currents, and demographic expansion. We propose that freshwater discharge from the Yangtze River, which may act as physical barrier limiting the southward dispersal of larvae from northern populations, is especially important in determining the separation of the YS group from the rest of the Chinese populations of *C. toreuma*.

## Introduction

Understanding the forces which contribute to the biogeographic distributions of marine organisms has long been of interest to biologists in their attempts to interpret present day patterns of marine biodiversity [1], [2]. Species in the intertidal zone are excellent candidates to study the influence of various factors on biogeographic distribution as they occupy a discrete, narrow strip of habitat which is linked by the sea [3], and so it is relative easy to identify the factors which affect their distribution. The distributions of intertidal species are closely linked to historic events, affecting the connectivity of different regions [4]–[7], present day coastal topography and oceanic currents which affect larval supply [8]–[11], [12], [13], species' ecological requirements [7], [14] and also present day changes in climate which influence the on-shore success of populations [15]–[17].

Fossil and pollens records have shown that historical climate change is a pivotal factor affecting the abundance and distribution of species [18], [19], and genetic data also confirm that historic events have had a significant impact on the distribution of populations [20]–[24], especially during the last glacial maximum (LGM) [18], [25], [26]. Previous studies have, for example, shown that the LGM did not extirpate the majority of species in the northeastern Pacific; instead, many species persisted in regional glacial refuges during the LGM, and this appears to be a common biogeographic history for many rocky-shore organisms in this region [6], [7].

**Figure 1:**
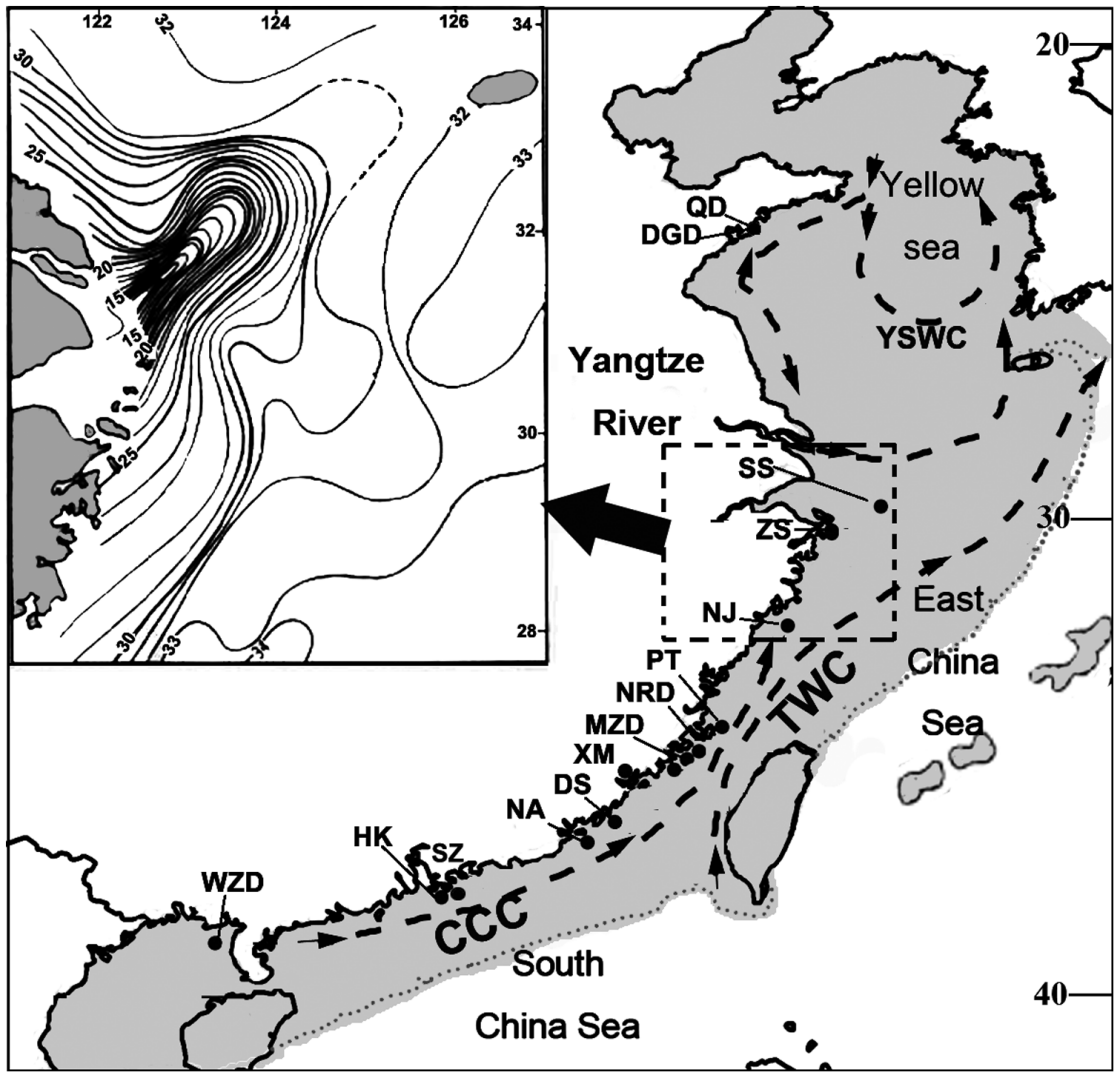
Collection sites and environmental conditions. Collection sites for *Cellana toreuma* and summer ocean currents along the China coast. CCC, China Coastal Current; YSWC, Yellow Sea Warm Current; TWC, Taiwan Warm Current [55]. The shaded area represents the estimated land area during the LGM [45]. Inset: Sea surface salinity in Yangtze River Estuary in summer [55].

As many intertidal species have planktonic larvae which can be carried in the water column before settlement for days or weeks [27]–[29], this larval dispersal phase is another important regional process affecting the distribution of intertidal species [30]–[32]. The dispersal distances of many marine organisms are directly related to the time that larvae spend in the plankton [29], however, the pelagic larval durations (PLD) among organisms are species, season and location specific, and dispersal distance is closely related to local and regional hydrographic conditions, biological characteristics [11], and habitat specificity [33], [34].

Limpets are important keystone grazers on rocky shores [35]–[37] and historic events have been shown to play a critical role in structuring the gene pool of patellid limpets from the temperate Northern Hemisphere [38]–[41]. Historic events have also been implicated as the main driver to explain the biogeography of nacellid limpets in New Zealand, with the formation of a geographic barrier by the Cook Strait being important for the current day biogeographic patterns observed in this group [23], [42], [43]. Maintenance of population connectivity through larval dispersal has also been shown to be important, the main ocean currents flowing along the Atlantic coast of Iberia have, for example, been responsible for maintaining connectivity of populations of *Patella rustica* along the coast through occasional larval transport [44]. Therefore, the complicated biogeographic patterns of limpets can serve as a model for understanding the present day distribution patterns of intertidal species along heterogeneous coastlines.

The distribution patterns of intertidal species along the coast of China are poorly studied but are likely to be influenced by past historic events as well as present day environmental conditions. The Bo, Yellow and East China Sea along the continental shelf of China, for example, are shallow seas and when global sea levels fell during the Quaternary glaciations, these three seas are thought to have become partly continuous landmasses (Fig. 1). Sea surface temperatures (SST) also decreased during this period [45] and such environmental changes are suggested to have affected species' distributions [46]–[50]. Besides historical events, ocean currents also play an important role in population connectivity along the China coast. The biogeography of a high shore barnacle, *Hexechamaesipho pilsbryi* (Hiro, 1936), for example, has been shown to be strongly influenced by the Kuroshio Current [47]. Hydrographic patterns can aslo be affected by outflows from major rivers [51]. Flow from the Yangtze River, the third largest river in the world with an average annual discharge of 8∼9×10^11^ m^3^ [52], can influence surrounding ocean currents [53], and may subsequently limit dispersal of planktonic larvae and act as a barrier for genetic connectivity of intertidal species, and hence play an important role in the biogeographic pattern of intertidal communities.

**Table 1:** Collection sites, sample size, allocated geographic group and summary of molecular diversity for *Cellana toreuma* collected along the coast of China.

ID	Site	Sample size	No. of haplotypes^1^	Nucleotide diversity (π, Mean ± S.D.)	Gene diversity (*H*, Mean ± S.D.)	Group^2^
QD	Qingdao (36°07′N, 120°36′E)	21	3 (71% 23%*)	0.00080±0.00081	0.45±0.10	YS
DGD	Dagongdao (36°02N′ 120°13′E)	30	5 (67% 23%*)	0.00096±0.00089	0.51±0.08	YS
SS	Shengshan (30°07′N 121°55E)	15	3 (86%)	0.00022±0.00040	0.26±0.14	ECS
ZS	Zhoushan (29°57′N 122°12′E)	52	5 (90%)	0.00032±0.00046	0.18±0.07	ECS
NJ	Nanjiliedao (27°33′N 120°41′E)	30	5 (86%)	0.00056±0.00064	0.25±0.10	ECS
PT	Pingtan (25°27′N 119°48′E)	29	3 (93%)	0.00023±0.00039	0.14±0.08	ECS
NRD	Nanridao (25°14′N 119°27′E)	32	4 (90%)	0.00032±0.00046	0.18±0.09	ECS
MZD	Meizhoudao (25°14′N 119°09′E)	24	4 (87%)	0.00028±0.00044	0.24±0.11	ECS
CW	Chongwu (24°53′N 118°56′E)	32	4 (91%)	0.00032±0.00046	0.18±0.09	ECS
XM	Xiamen (24°25′N 118°08′E)	32	5 (87%)	0.00042±0.00054	0.25±0.10	ECS
DS	Dongshan (23°43′N 117°32′E)	32	2 (97%)	0.00011±0.00026	0.06±0.06	ECS
NA	Nanao (23°26′N 117°00′E)	30	4 (90%)	0.00034±0.00048	0.19±0.10	ECS
SZ	Shenzhen (22°32′N 114°13′E)	14	2(86% 14%**)	0.00044±0.00058	0.26±0.14	SCS
HK	Hong Kong (22°17′N 144°10′E)	31	3 (90%)	0.00032±0.00046	0.19±0.09	SCS
WZD	Weizhoudao (21°26′N 109°03′E)	14	2 (92%)	0.00048±0.00061	0.14±0.12	SCS

1 Values in the parentheses are the percentages of haplotype 1 in the populations;

* percentages of haplotype no. 2 in QD and DGD populations;

** percentages of haplotype no. 10 in SZ populations.

2 Based on the geographical locations, these populations were divided into three groups. YS, ECS and SCS represent populations from Yellow Sea, East China Sea and South China Sea, respectively.

The limpet *Cellana toreuma* (Reeve, 1855) is widely distributed along the China coast [54], which makes it an ideal species to investigate the relative roles of historic events and present day environmental conditions on the past and present genetic connetivities of interidal species in this region. Specifically, phylogenetic studies of 15 populations of *C. toreuma* from Qingdao in the north, to Weizhoudao in the south of China were used to investigate the importance of historic events, the role of ocean currents and Yangtze River discharge in affecting the present day genetic connectivity of intertidal, rocky shores species along the China coast.

## Materials and Methods

### Collections

A total of 418 individuals of C*ellana toreuma* from 15 rocky shore localities which represented populations from different marginal seas were collected in 2009–2010 within a geographic range of ∼2,600 km along the coastline of China (Fig. 1 and Table 1). The summer ocean currents along China coast and sea surface salinity in Yangtze River Estuary in summer [55] were shown in Fig. 1. Based on their locations, the 15 populations could be divided into three groups, the Yellow Sea group (YS group, including QD and DGD), the East China Sea group (ECS group, including SS, ZS, NJ, PT, NRD, MZD, CW, XM, DS and NA) and the South China Sea group (SCS group, including SZ, HK and WZD). As *C. toreuma* is not a protected species, and collections were only made from public access areas, no specific permits were required to collect this species from these locations/activities.

### DNA extraction, PCR and sequencing

All individuals were stored in absolute ethanol until DNA extraction. Genomic DNA was extracted from foot muscle tissue using methods as described in previous studies [56] with minor revisions. About 20 mg of foot muscle was dissected from each individual and homogenized in 400 µl lysis buffer (400 mM NaCl, 10 mM Tris-HCl (pH8.0), 2 mM EDTA (pH 8.0), 1% SDS (w/v). 10 µl (10 mg ml^−1^) of protease K was added to the homogenate, which was then incubated in a water bath at 55°C. After 1∼3 h incubation, 400 µl NaCl (6 M) was added and the homogenate was centrifuged at 12,000 *g* for 30 min. An equal volume of isopropanol was added to the supernatant and then the mixture was centrifuged at 12000 *g* for 15 min. The precipitate was washed using 70% ethanol twice and then dissolved in ultrapure water. A 598 nucleotide fragment of cytochrome oxidase subunit I mtDNA (COI) was amplified and sequenced using the following primers: LCO1490F5′- GGT CAA CAA ATC ATA AAG ATA TTG G -3′, HCO2198R 5′- TAA ACT TCA GGG TGA CCA AAA AAT CA -3′ [57].

### Data analyses

Sequences of each individual were aligned with Clustal X1.81 [58] and individual consensus sequences were retrieved with both alignment and manual checks. The accuracy of COI sequences was confirmed by translating the nucleotide data to amino acid sequences. Estimates of molecular genetic diversity (π, nucleotide diversity; H, gene diversity; F_ST_ values, and haplotype frequencies) were performed using Arlequin 3.5 [59]. As sample sizes varied between different populations and groups, the programme Contrib 1.02 was used to standardized samples sizes, and haplotype frequencies were then calculated to estimate gene diversity and divergence of populations and groups [60].

Analysis of molecular variation (AMOVA) [61], performed in Arlequin, was used to examine population genetic structure. Historical demographic expansions were examined by Tajima's D test [62] and Fu's *F*s [63]. Historic demographic expansions were also investigated by examination of frequency distributions of pairwise differences between sequences (mismatch distribution) [61]. Neutrality and mismatch distribution tests were also performed in Arlequin.

Phylogenetic trees of haplotypes were constructed using Mega 5.0 [64]. Neighbour-joining (NJ) tree was reconstructed using evolutionary distances computed with the Maximum Composite Likelihood method using the Tamura-Nei substitution model. To examine the relationship between genetic and geographic distance, the pairwise values of F_ST_ were calculated against geographic distance between all locations. The strength and significance of the relationship between genetic differentiation and geographic distances were assessed with Mantel tests using Ibd 1.53 [65]. To visualize the relationship between genetic and geographic distances, pairwise population F_ST_ values were plotted against geographic distance using Prism v5.0 (Graphpad Software, San Diego, CA, USA).

## Results

### Sequence variations

A 597-bp portion of COI was sequenced from 418 individuals of *Cellana toreuma* (GenBank access numbers JQ313140-JQ313557). Among all the individuals, 30 nucleotide sites were polymorphic. The genetic variation of COI mtDNA in the 15 populations of *Cellana toreuma* along the China coast was low, whether measured as gene diversity (H, 0.06±0.06 to 0.51±0.08) or nucleotide diversity (π, 0.00011±0.00026 to 0.00096±0.00089, Table 1). The two populations from the Yellow Sea had relatively high genetic differences (QD: H, 0.45; π, 0.00080; DGD, H, 0.51; π, 0.00096) as compared to the populations from the East China Sea and South China Sea (Table 1). Results calculated using CONTRIB were similar, with the highest diversity also observed in DGD (DHs = 0.362; DHt = 0.404) and QD (DHs = 0.333; DHt = 0.087, Table 2).

**Table 2:** Measures of genetic diversity and divergence for 15 population and three groups based on haplotype frequencies using CONTRIB 1.02 (see Table 1 for site and group abbreviations).

	Samplesize	Nb Hap	*h*	*h_o_*	r(13)	DH_s_	DH_t_	DG_st_	C_t_	C_s_	C_d_	C^r^_t_	C^r^_s_	C^r^_d_
Population levels														
CW	32	4	0.181	0.090	1.219	0.207	0.211	0.018	−0.017	−0.014	−0.003	−0.111	−0.004	−0.106
DG	30	5	0.515	0.089	2.290	0.362	0.404	0.104	0.108	0.086	0.022	−0.008	0.046	−0.054
DS	32	2	0.063	0.058	0.406	0.152	0.160	0.056	−0.05	−0.050	0.000	−0.155	−0.042	−0.113
SS	15	3	0.257	0.142	1.733	0.242	0.242	0.002	0.003	0.009	−0.005	−0.062	0.02	−0.081
ZS	52	5	0.184	0.072	1.191	0.208	0.213	0.022	−0.016	−0.013	−0.002	−0.106	−0.006	−0.100
NJ	30	5	0.253	0.104	1.733	0.24	0.242	0.010	0.003	0.007	−0.004	−0.085	0.02	−0.105
NR	32	4	0.181	0.09	1.219	0.207	0.211	0.018	−0.017	−0.014	−0.003	−0.111	−0.004	−0.106
HK	30	3	0.131	0.082	0.867	0.183	0.189	0.029	−0.031	−0.029	−0.002	−0.127	−0.021	−0.106
WZ	14	2	0.143	0.119	0.929	0.189	0.193	0.020	−0.029	−0.026	−0.003	−0.098	−0.018	−0.080
XM	32	5	0.238	0.099	1.625	0.233	0.236	0.013	−0.001	0.003	−0.003	−0.087	0.015	−0.102
SZ	14	2	0.264	0.136	1.000	0.245	0.250	0.021	0.008	0.011	−0.002	−0.062	−0.015	−0.047
MZ	24	4	0.239	0.113	1.625	0.234	0.236	0.009	−0.001	0.003	−0.004	−0.09	0.015	−0.105
PT	29	3	0.135	0.085	0.897	0.185	0.190	0.026	−0.030	−0.028	−0.002	−0.126	−0.019	−0.106
QD	21	3	0.452	0.105	1.616	0.333	0.364	0.087	0.082	0.067	0.015	−0.028	0.014	−0.042
NA	30	4	0.193	0.095	1.300	0.212	0.216	0.016	−0.014	−0.011	−0.003	−0.106	0.000	−0.105
Group level														
YS group	51	5	0.481	0.068	3.619	0.344	0.388	0.115	0.372	0.279	0.093	−0.004	−0.029	0.025
ECS group	295	25	0.193	0.031	4.135	0.272	0.295	0.077	−0.201	−0.160	−0.041	0.035	0.025	0.010
SCS group	43	5	0.220	0.083	3.930	0.279	0.300	0.070	−0.170	−0.119	−0.052	−0.104	0.004	−0.108

### Genetic relationship among haplotypes

**Figure 2:**
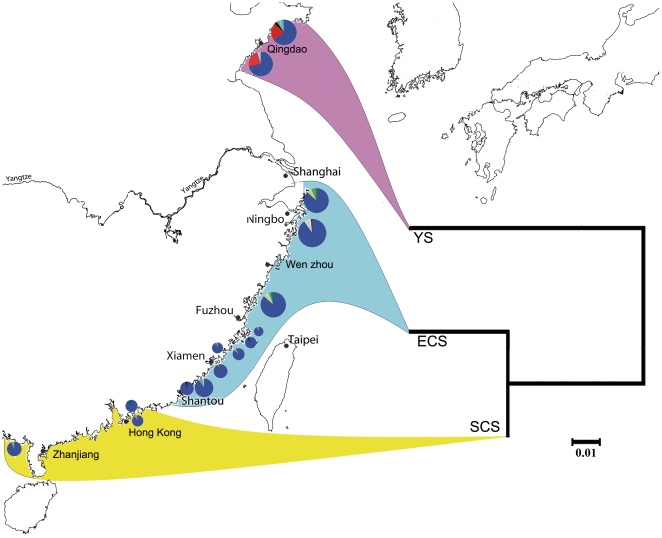
Haplotype frequencies of COI mtDNA. Haplotype frequencies of partial COI mtDNA from 15 populations of *Cellana toreuma*. In each circle, blue and red represent the proportions of H1 and H2, respectively. Inset: Neighbour-joining tree of partial COI mtDNA of the three groups (see Table 1 for population code).

Of the 418 individuals from 15 populations, 30 haplotypes were present (see Appendix S1). A dominant haplotype No. 1(H1) was found in all the populations (*ca.* 87% of individuals). The percentage of H1 in all populations ranged from 67–97%. In populations from the East China Sea, percentages of H1 in all the samples were >85% (86%–97%). All other haplotypes differed from H1 by only one or two mutations. In the two populations from the Yellow Sea (QD and DGD), however, the percentages of haplotype No. 2 (H2) were both 23%, indicating H2 was an important private haplotype in these populations (Table 1, Fig. 2). Haplotype relationships based on NJ tree revealed no significant genealogical branches or clusters of samples corresponding to sampling locality (see Appendix S2).

### Population genetics and demography

Genetic differentiation among different populations was evaluated using the genetic distances of Tajima-Nei values (see Appendix S3). In general, pairwise distances were low (−0.0387∼0.18509). The largest genetic differentiation occurred between QD and DS populations (0.18509, P<0.001). The pairwise distances were negative (−0.0387, P = 0.99) for the two populations from the Yellow Sea (QD and DGD), indicating that the variation within populations was greater than the variation between populations. In the 10 populations from the East China Sea, the pairwise distances were also very low (−0.01394 to 0.00514) and insignificant (P>0.05), as was the case for the three populations from the South China Sea (SZ, HK and WZD, 0.0387∼0. 01856, P>0.05). The divergence of the populations calculated using CONTRIB showed that the absolute gene differentiation was largest for the most remote, isolated population, DGD (DGst = 0.104) followed by the other most isolated population, QD (DGst = 0.104, Table 2).

F_ST_ values among the three groups (the Yellow, East China and South China Sea), were low (0.00613 to 0.18991, Table 3). The F_ST_ value between the East China and South China Sea was 0.00613, whilst the F_ST_ values between the Yellow Sea and the other two groups were relatively high (East China Sea, F_ST_ = 0.18991, P<0.001; South China Sea, F_ST_ = 0.12820, P<0.001). The absolute gene differentiation (DGst) calculated using CONTRIB was highest in the YS group (0.115) as compared to the two other groups (ECS, 0.077; SCS, 0.070, Table 2). AMOVA analysis showed genetic differences among groups (F_CT_), genetic differences among populations within groups (F_SC_) and genetic differences among populations (F_ST_, Table 4), indicating the existence of relatively high genetic differences among all different group levels.

**Table 3:** Pairwise genetic distances between populations of *Cellana toreuma* separated into the Yellow Sea, East China Sea and South China Sea groups along the China coast^1^.

	YS group	ECS group	SCS group
YS group		0.18991	0.12820
ECS group	<0.000001		0.00613
SCS group	<0.000001	0.07207	

1 The upper matrix shows the Tajima-Nei F_ST_ values and the lower matrix shows the P values.

**Table 4:** Analysis of molecular variation for samples of *Cellana toreuma* along the China coast to investigate genetic differences in different levels.

Source of variation	d. f.	Sum of squares	Variance components	Percentage of Variation	Fixation Index	P value
Among Groups	2	2.753	0.01439	10.90	0.10901	0.00000
Among populations within groups	12	1.263	−0.00046	−0.35	−0.00391	0.51026
Within populations	403	47.53	0.11809	89.45	0.10553	0.00000
Total	417	51.604	0.13202			

**Figure 3:**
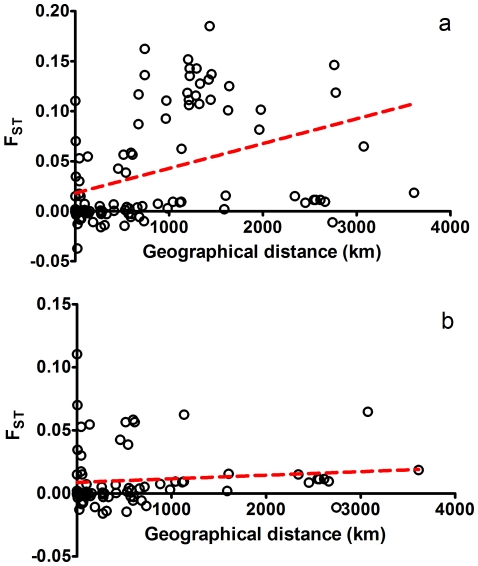
Relationship between genetic distance and geographic distance. (A) Relationship between genetic distance and geographic distance for all populations; (B) Relationship between genetic distance and geographic distance for populations from the East China Sea and South China Sea groups.

The Mantel test indicated a significant relationship (P = 0.046) between F_ST_ and geographic distance in all 15 populations, indicating evidence of isolation by distance, with geographic distance explaining 15% of the variation in genetic differentiation between populations of *Cellana toreuma* (*r* ^2^ = 0.15). However, such a relationship between genetic variation and geographic difference was not found among populations from the East China or South China Sea groups (P = 0.192), with only ∼1% of the variation in genetic differentiation being explained by geographic distance (*r* ^2^ = 0.01, Fig. 3).

**Figure 4:**
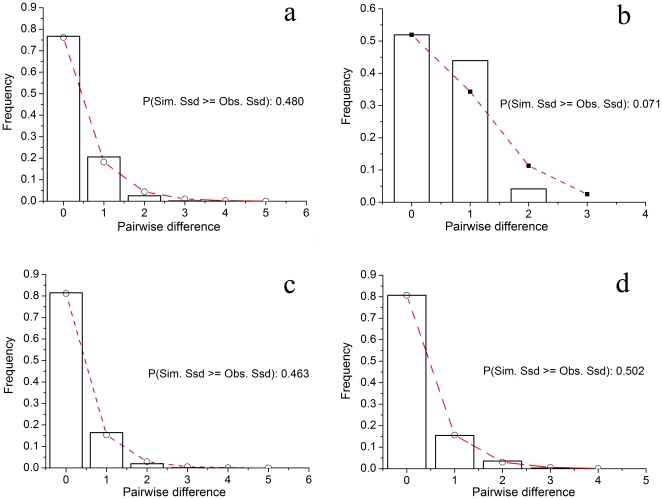
Observed pairwise differences and expected mismatch distribution. Observed pairwise differences (bars) and the expected mismatch distributions (line) under the sudden expansion model of COI gene. (A) in all samples, (B) in Yellow Sea group, (C) in East China Sea group and (D) in South China Sea group.

Tajima's D and Fu's *F*s were used to test for neutrality, and the results in different locations were variable (Table 5). All Tajima's D-values were negative. At the population level, populations from the Yellow Sea had insignificant negative values as did populations from the South China Sea (SZ, HK and WZD, Table 5). In contrast, all populations from the East China Sea showed significant negative values indicating significant population expansion of *C. toreuma* in the East China Sea (Table 5). Mismatch distributions, to explore the distribution of the number of pairwise differences between haplotypes, were unimodal, matching the sudden expansion model (Fig. 4).

**Table 5:** Tajima's D and Fu's Fs, and corresponding P values, and mismatch distribution parameters for 15 populations of *Cellana toreuma* and their associated groups along the China coast (see Table 1 for site and group abbreviations).

ID	Tajima's D		Fu's *F*s		Mismatch distribution		
	D	P	*F*s	P	τ	θ_0_	θ_1_
Population level							
QD	−0.33029	0.31900	−0.27034	0.28100	0.60938	0	99999.00000
DGD	−1.11781	0.01560	−2.13140	0.04300	0.71875	0	99999.00000
SS	−1.15945	0.17200	−1.54636	0.02000	3.00000	0	0.36533
ZS	−1.76473	0.01300	−4.92636	0.00000	3.00000	0	0.23760
NJ	−2.00763	0.00200	−3.14992	0.00200	3.00000	0	0.32139
PT	−1.50906	0.01500	−2.31203	0.00600	3.00000	0	0.16396
NRD	−1.72954	0.01400	−3.48995	0.00000	3.00000	0	0.23271
MZD	−1.51469	0.01500	−3.02081	0.00100	3.00000	0	0.33115
CW	−1.72954	0.01400	−3.48995	0.00100	3.00000	0	0.23271
XM	−1.88876	0.01000	−4.54522	0.00000	3.00000	0	0.32871
DS	−1.14244	0.12000	−1.26483	0.05300	3.00000	0	0.06855
NA	−1.73178	0.01300	−3.38072	0.00000	3.00000	0	0.25225
SZ	−0.34144	0.20100	0.18574	0.30400	0.33008	0	99999.00000
HK	−1.26161	0.06800	−1.70822	0.01400	3.00000	0	0.24004
WZD	−1.48074	0.07300	0.29648	0.34900	3.00000	0	0.10537
Total							
Group level							
YS group	−0.93178	0.01830	−1.86278	0.07700	0.66016	0	99999.00000
ECS group	−2.48839	0.00000	−∞	0.00000	3.00000	0	0.23271
SCS group	−1.83152	0.00400	−∞	0.00100	3.00000	0	0.24004
Total					0.672	0	99999.00000

## Discussion

A dominate haplotype (H1) was found to be ubiquitously distributed in all the studied populations of *Cellana toreuma* along the China coast. The high percentages and wide distribution of H1 in the 15 sampled populations indicates that a population expansion of this species has occurred. Evidence for the occurrence of a population expansion was supported by the significant negative values of the neutrality tests (Tajima's D test and Fu's *F*s test), especially in the East China Sea populations. The unimodal mismatch distribution was also in accordance with an expected sudden population expansion [66]. These results, therefore, support the hypothesis that *C. toreuma* populations along the China coast had experienced a large-scale population expansion.

The population expansions and contractions were closely associated with Pleistocene-era environmental fluctuations [6]. The glacial and inter-glacial exchanges have had significant impacts on current phylogeographic patterns [18]. During the last glacial maximum (12,000 and 75,000 years ago), sea level decreased dramatically in the marginal seas of China (∼100–120 m in the South China Sea, and 130–150 m in the East China Sea [45]). As a consequence of sea level dropping, the East China Sea was reduced to an elongated trough, the Okinawa Trough, while the South China Sea became a semi-enclosed gulf [45]. Due to the southern shift of the polar front in the North Pacific, and the reorganization of the surface current systems, winter temperatures in the South China Sea were ∼6–10°C colder than the present day, and seasonality was much stronger [45]. As the environmental conditions in the intertidal zone are affected by both marine and terrestrial factors, intertidal species experience stronger environmental stresses than animals living in the ocean [6]. The cold and dry conditions during the LGM would, therefore, be expected to eradicate most *Cellana toreuma* in northern China and can explain the post-LGM recolonization from southern to northern China after the LGM. The genetic connectivity of *C. toreuma* along the China coast can, therefore, be explained by a demographic expansion which may have been mediated by transport of larvae via the Kuroshio Current, China Coastal Current and the Yellow Sea Warm Current (see Fig. 1).

The relative higher nucleotide and gene diversity in the Yellow Sea group indicates that there are other important factors affecting the phylogeographic pattern of *Cellana toreuma* along the China coast. As described in Hewitt [67], low genetic diversity at relatively high latitudes, with a small number of alleles or haplotypes dominating disproportionately large areas, is a pattern most consistent with the hypothesis of a recent range extension from a southern refuge. In the present study, however, the nucleotide diversity and gene diversity in QD and DGD were higher than those of the southern populations, and haplotype No. 2 (H2) was common in the Yellow Sea group, but could not be found in populations from the East China or South China Seas. The pairwise genetic distance between the Yellow Sea group and the other two groups were relatively high, and AMOVA analysis suggested the presence of geographic structure in the populations. The Yellow Sea group was, therefore, relatively isolated from the other two groups. Three principal factors may potentially contribute to the observed geographic structure of *C. toreuma* populations along the China coast: firstly populations survived in northern refuges during the LGM or, secondly, the existence of large areas of unsuitable habitat can form barriers that potentially isolated the Yellow Sea populations, or finally, that the observed genetic isolation is due to the effect of contemporary ocean currents and/or Yangtze River discharge.

There was only one mutational step between H1 and H2, and the genetic distance between the two haplotypes was very low, indicating the isolation of H2 was a relatively contemporary event. In southern Australia, a clear phylogeographic break in the distribution of the barnacle, *Catomerus polymerus*, was found due to the large expanse of sandy shores devoid of suitable rocky substrate for *C. polymerus* to settle on along Ninety Mile Beach [68]. The coastline between Qingdao and the Yangtze River is also composed of long stretches of unsuitable habitat for *C. toreuma* (∼400 km), such as salt marshes, which might act as possible barriers restricting the dispersal of *C. toreuma* larvae. In this region, however, there are intermittent islands, dams and harbours, which may provide suitable hard substrates for colonization by *C. toreuma* and act as stepping-stones for colonization, as has been described for rocky intertidal invertebrates in south-eastern Australia [69]. It seems unlikely, therefore, that there is a significant barrier caused by a lack of suitable substrata which is isolating the Yellow Sea populations, although this requires further confirmation.

The relatively higher nucleotide and gene diversities in the Yellow Sea group are, therefore, most likely related to population disjunctions, due to the impact of ocean currents and/or Yangtze River discharge. This explanation seems plausible as pelagic larval dispersal will be the major source of contemporary gene flow and hence distribution of *C. toreuma*. Reproduction in *C. toreuma* usually occurs in summer. In north Zhejiang, China, *C. toreuma* reproduces from June to September [70] and in Tanabe Bay, Japan, it breeds twice in April to June and September to October [71]. Although the exact length of the pelagic larval stage is unknown for this species, it varies between 4–18 days in congenerics [38]. In spring and summer, the plume of water from the Yangtze River discharge shifts in a northerly direction in parallel with the Taiwan warm current with a clockwise deflection (Fig. 1). The size and distance of deflection of the dilute water in summer is greater than in spring. As a result, the East China Sea coastal current, which flows from north to south along the coast in winter, is deflected at the mouth of the Yangtze River due to the increased river discharge in spring and summer [72]. The dilute plume of Yangtze River waters in spring and summer can also cause a decrease in the salinity of the upper layer of the Kuroshio Current [73], which will affect nutrient concentrations [74], and subsequently phytoplankton biomass [75]. The disruption of the southward East China Sea coastal current due to the impact of the summer monsoon and discharge from the Yangtze River during the reproductive season of *C. toreuma* may cause a disconnection between the Yellow Sea populations and the southern populations of this limpet. This may, therefore, isolate the unique haplotype (H2) in the Yellow Sea group as larvae with this haplotype would be unable to disperse south to the East China Sea and subsequently South China Sea populations of *C. toreuma*.

The phylogeographic pattern of *Cellena toreuma* is different from that seen in the alga, *Sargassum horneri* [76]. *S. horneri* populations have good genetic connectivity with no apparent physical barriers to dispersal along the China coast. It has been suggested that the China Coastal Current in autumn and winter can transport floating marine organisms, such as fragments of *Sargassum*, along the China coast from the Yellow and Bohai Sea to the East China Sea [76]. As *S. horneri* mature twice a year (in spring and autumn), the southward China Coast Current in winter can transport their propagules from the Yellow sea to East China Sea, and then to the South China Sea, and will not be affected by the barrier created by the summer Yangtze discharge. In contrast to *S. horneri*, the pelagic larval dispersal stage of *C. toreuma* is limited to only 4–18 days. Most importantly, the reproductive season of *C. toreuma* occurs only during summer, when the southward China Coast Current is interrupted by freshwater discharge from the Yangtze River which decreases salinity significantly, and therefore acts as a temporary barrier to the demographic expansion for northern *C. toreuma* populations. The phylogeographic patterns of intertidal species along the China coast are, therefore, closely related to species-specific life cycles and related ecological characteristics.

In general, therefore, both historic events and contemporary oceanographic conditions play important roles in the demographic expansion of the intertidal limpet, *Cellana toreuma*. Along the coast of China the interplay between these factors is strongly influenced by the timing of intense discharge from the Yangtze River. The coincidence of increased Yangtze River discharge and the resulting weakening of the China Coast Current in summer, when larvae of *C. toreuma* will be in the water column, act as a physical barrier, inhibiting the southern dispersal of limpet larvae and playing an important role in the phylogeographic patterns of this limpet along the China coast.

## Supporting Information

Appendix S1 The haplotypes frequencies of COI in different *Cellana toreuma* populations in China coast.
(DOC)

Appendix S2 Neighbour-joining tree based on COI dataset with bootstrap values of 1,000 above each branch of interest with other *Cellana* limpets as outgroups.
(PDF)

Appendix S3 Genetic pairwise distances of the COI mitochondrial gene between 15 *Cellana toreuma* populations.
(DOC)
