# Supplementary figures and images for: The Impact of Yangtze River Discharge, Ocean Currents and Historical Events on the Biogeographic Pattern of Cellana toreuma along the China Coast
Source: PLoS One. 2012 Apr 26;7(4):e36178. doi: 10.1371/journal.pone.0036178 (PMC3338569; doi:10.1371/journal.pone.0036178)

NJ

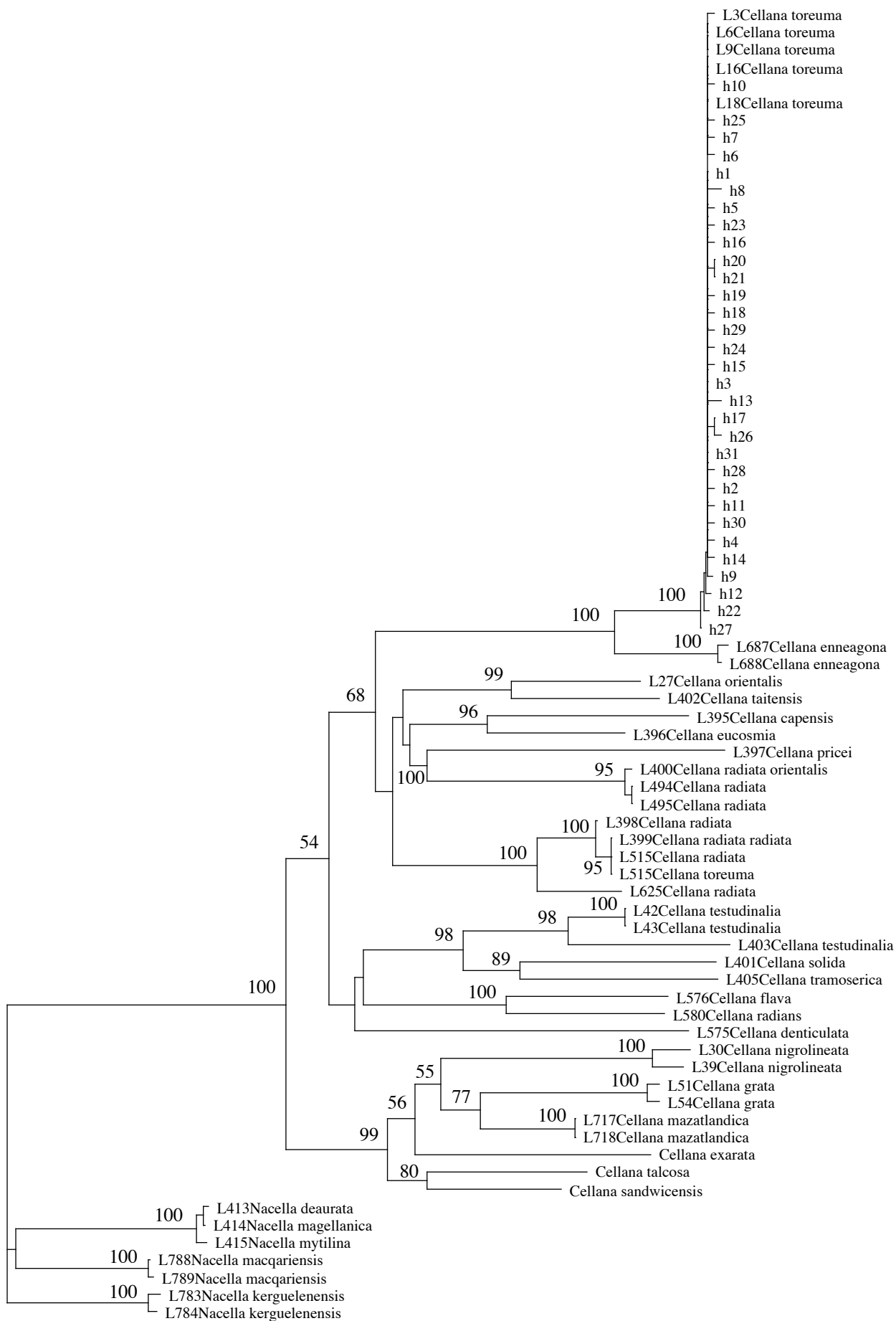

Supplement: Appendix S2 — Neighbour-joining tree based on COI dataset with bootstrap values of 1,000 above each branch of interest with other Cellana limpets as outgroups. (PDF) [file pone.0036178.s002.pdf]
