# Supplementary material for: The Impact of Yangtze River Discharge, Ocean Currents and Historical Events on the Biogeographic Pattern of Cellana toreuma along the China Coast
Source: PLoS One. 2012 Apr 26;7(4):e36178. doi: 10.1371/journal.pone.0036178 (PMC3338569; doi:10.1371/journal.pone.0036178)
Supplement: Appendix S3 — Genetic pairwise distances of the COI mitochondrial gene between 15 Cellana toreuma populations. (DOC) [file pone.0036178.s003.doc]

**Appendix S3** Genetic pairwise distances of the COI mitochondrial gene between 15 *Cellana toreuma* populations^1^

|  | QD | DGD | NA | PT | NRD | MZD | XM | NJ | DS | CW | SS | ZS | SZ | HK | WZD |
| --- | --- | --- | --- | --- | --- | --- | --- | --- | --- | --- | --- | --- | --- | --- | --- |
| QD |  | 0.99099 | 0.00000 | 0.00000 | 0.00000 | 0.00901 | 0.00901 | 0.00901 | 0.00901 | 0.00901 | 0.02703 | 0.00000 | 0.04505 | 0.00000 | 0.01802 |
| DGD | -0.03721 |  | 0.00000 | 0.02703 | 0.00901 | 0.00901 | 0.00000 | 0.00901 | 0.00000 | 0.00000 | 0.09009 | 0.00000 | 0.00901 | 0.00000 | 0.06306 |
| NA | 0.13707 | 0.11141 |  | 0.90991 | 0.6036 | 0.76577 | 0.71171 | 0.8018 | 0.29730 | 0.81982 | 0.63063 | 0.27027 | 0.13514 | 0.85586 | 0.36937 |
| PT | 0.15179 | 0.11826 | -0.01394 |  | 0.66667 | 0.63964 | 0.84685 | 0.84685 | 0.52252 | 0.73874 | 0.51351 | 0.28829 | 0.14414 | 0.88288 | 0.50450 |
| NRD | 0.14272 | 0.11117 | 0.00012 | -0.00044 |  | 0.62162 | 0.26126 | 0.27928 | 0.99099 | 0.35135 | 0.40541 | 0.57658 | 0.00000 | 0.13514 | 0.31532 |
| MZD | 0.13526 | 0.10622 | -0.00073 | 0.00073 | -0.00056 |  | 0.63964 | 0.77477 | 0.37838 | 0.65766 | 0.64865 | 0.40541 | 0.15315 | 0.23423 | 0.30631 |
| XM | 0.12764 | 0.10726 | -0.00019 | -0.00091 | 0.00005 | -0.00201 |  | 0.41441 | 0.76577 | 0.55856 | 0.56757 | 0.27928 | 0.12613 | 0.21622 | 0.30631 |
| NJ | 0.11063 | 0.09257 | -0.00837 | -0.00049 | 0.00063 | -0.00271 | 0.00037 |  | 0.16216 | 0.66667 | 0.68468 | 0.21622 | 0.14414 | 0.29730 | 0.77477 |
| DS | 0.18509 | 0.13158 | 0.00110 | 0.00128 | -0.01591 | 0.00514 | -0.00004 | 0.00136 |  | 0.8018 | 0.58559 | 0.44144 | 0.05405 | 0.18919 | 0.27928 |
| CW | 0.14273 | 0.11559 | -0.01077 | -0.00044 | 0.00005 | -0.00059 | 0.00004 | -0.00750 | -0.00002 |  | 0.51351 | 0.27928 | 0.0991 | 0.25225 | 0.31532 |
| SS | 0.11679 | 0.08709 | -0.00633 | -0.00046 | -0.00569 | -0.00272 | -0.00991 | -0.01285 | 0.01497 | -0.00569 |  | 0.45946 | 0.16216 | 0.28829 | 0.36937 |
| ZS | 0.16224 | 0.13612 | 0.00426 | 0.00205 | -0.00243 | 0.00265 | 0.00525 | 0.00730 | -0.00020 | 0.00391 | -0.00346 |  | 0.06306 | 0.13514 | 0.20721 |
| SZ | 0.12501 | 0.10088 | 0.05286 | 0.07006 | 0.05656 | 0.05843 | 0.04264 | 0.03004 | 0.11045 | 0.05654 | 0.05470 | 0.06251 |  | 0.12613 | 0.21622 |
| HK | 0.14621 | 0.11856 | -0.01111 | -0.01451 | 0.01137 | 0.01131 | 0.00960 | 0.00855 | 0.01744 | 0.01137 | 0.00687 | 0.01506 | 0.06478 |  | 0.31532 |
| WZD | 0.10158 | 0.08150 | 0.00752 | 0.01510 | 0.00944 | 0.00883 | 0.00301 | -0.00277 | 0.03463 | 0.00943 | 0.00202 | 0.01554 | 0.03870 | 0.01856 |  |

^1^ The lower matrix shows the Tajima-Nei values and the upper matrix shows the P values
